# Sponge-Like: A New Protocol for Preparing Bacterial Ghosts

**DOI:** 10.1155/2013/545741

**Published:** 2013-03-18

**Authors:** Amro A. Amara, Mounir M. Salem-Bekhit, Fars K. Alanazi

**Affiliations:** ^1^Department of Protein Research, Genetic Engineering and Biotechnology Research Institute, Mubarak City for Scientific Research and Technology Applications, Alexandria, Egypt; ^2^Department of Pharmaceutics, College of Pharmacy, King Saud University, P.O. Box 2457, Riyadh 11451, Saudi Arabia; ^3^Department of Pharmaceutics, Kayyali Chair for Pharmaceutical Industries, College of Pharmacy, King Saud University, P.O. Box 2457, Riyadh, Saudi Arabia; ^4^Department of Microbiology and Immunology, Faculty of Pharmacy, Al-Azhar University, Cairo, Egypt

## Abstract

Bacterial Ghosts (BGs) received an increasing interest in the recent years for their promising medicinal and pharmaceutical applications. In this study, for the first time we introduce a new protocol for BGs production. *E. coli* BL21 (DE3) pLysS (Promega) was used as a model to establish a general protocol for BGs preparation. The protocol is based on using active chemical compounds in concentrations less than the Minimum Inhibition Concentration (MIC). Those chemical compounds are SDS, NaOH, and H_2_O_2_. Plackett-Burman experimental design was used to map the best conditions for BGs production. Normal and electronic microscopes were used to evaluate the BGs quality (BGQ). Spectrophotometer was used to evaluate the amount of the released protein and DNA. Agarose gel electrophoresis was used to determine the existence of any residue of DNA after each BGs preparation. Viable cells, which existed after running this protocol, were subjected to lysis by inducing the lysozyme gene carried on pLysS plasmid. This protocol is able to produce BGs that can be used in different biotechnological applications.

## 1. Introduction

The BGs is the bacterial envelope without its internal content. Recently the common method, used for BGs preparation, is based on a cloned lysis gene *E *(a bacteriophage *ΦX174* gene). *E* lysis gene is lysis *E. coli* after bacteriophage infection [1]. To avoid pathogenic islands and antibiotic resistance genes in the BGs vaccine preparation, the DNA is degraded by a nuclease [2]. There are many existed applications for BGs. The live attenuated and inactivated whole-cell bacteria are an effective delivery system for recombinant antigens and nucleic acids [3]. Various animal models were used to immunize them against Gram-negative bacteria using BGs [4]. BGs can be used as vaccine candidates with intrinsic adjuvant properties based on well-known immune-stimulating compounds such as lipopolysaccharides, lipid A, and peptidoglycan. BGs can be used as a delivery system for protein/antigen, nucleic acids, drugs, and soluble compounds for various medical and technical applications [5, 6]. BGs cells were used recently as DNA delivery system by immobilizing the plasmid DNA [7]. The BGs system represents a new highly efficient gene delivery platform as an alternative to current viral and bacterial methods in vaccine development [8]. The inner space of BGs empty envelope can be loaded with a combination of peptides, drugs, or foreign DNA, which gives us an opportunity to design new types of poly-valent vaccines [9, 10]. In this study, we reinvestigate the possibility to establish a protocol for BGs preparation without the need for the *E* lysis gene. The protocol based on minimizing the effect of each of SDS, NaOH, CaCO_3_, and H_2_O_2_, which are used as an alternative for the *E* lysis gene to prepare the BGs. Plackett-Burman optimization and randomization method was used to map the proper quantities to be used and the best BGs preparation condition [11, 12]. 

## 2. Material and Methods

### 2.1. Bacterial Strain

*E. coli* recombinant cells BL21 (DE3) pLysS (Promega) was used in this study. The *E. coli* contains a plasmid pLysS. The *E. coli* genotype is F^−^, *omp*T, hsdS_B_(r_B_^−^,  m_B_^−^), *dcm*, gal, *λ* (DE3), pLysS, Cm^r^ [13, 14].

### 2.2. Bacterial Cultivation

The *E. coli* was cultivated in one liter flask contains 500 mL NB under static condition at 37°C for 72 hr.

### 2.3. Determination of the MIC and Minimum Growth Concentration (MGC) for NaOH, SDS, and H_2_O_2_

 Standard experiment for determining the MIC for each of NaOH, SDS, and H_2_O_2_ was conducted [15]. The MIC value for each compound was calculated as well as the concentration which allows first bacterial growth which is abbreviated as MGC (the concentration which showing first growth after the MIC).

### 2.4. Bacterial Ghost-Plackett-Burman Experiment

 The different variables were four chemical compounds representing SDS (X_1_), H_2_O_2_ (X_2_), CaCO_3_ (X_3_), and NaOH (X_4_), and two physical parameters were represented as one variable, temperature-shacking rate (X_5_). The five variables were randomized according to Plackett-Burman design as in Table 1. Twelve experiments following Plackett-Burman design were conducted to optimize the five used variables. Each variable was represented at two levels (high and low), which are donated by +1 and −1 as in Table 1. For each of SDS, H_2_O_2_, and NaOH the +1 value represent, the MIC while the −1 value represents the determined MGC. The biomass of the 72 h cultivated *E. coli* culture was collected by centrifuging the bacterial broth at 4000 rpm for 10 min. The cells then washed gently by 0.5% saline and recentrifuged at 4000 rpm for 10 min. The supernatant was then discarded. The *E. coli* cells were collected as a cells pellet and washed again. The cells concentration was equal to 10^6^ CFU/mL. 5x stock for each of NaOH, SDS, and H_2_O_2_ was prepared from both of the +1 and –1 values, which were determined from the MIC and the MGC as above. Twelve experiments were conducted following the design of the Plackett-Burman as in Table 1. Either –1 or +1 value of each variable was used according to the design. All the experiments were conducted in three steps. The first step contains all the variables except H_2_O_2_. After saline/water washing, the second step contains only H_2_O_2_. In the first step one ml of each of the 5x (+1 or −1) NaOH, SDS, and CaCO_3_ was added to 2 mL of the bacterial suspension to have a final volume equal to 5 mL and to give a final concentration equal to 1x for each. After 1 hr incubation according to the Plackett-Burman design, the different mixtures (for each experiment) were centrifuged each and the cells were collected as cell pellets using centrifugation at 4000 rpm for 10 min. The supernatant was then transferred to clean and sterile Falcon tubes. The cell pellets were then washed with 0.5% sterile saline and re-centrifuged. The supernatant then discarded and the cells suspended in 1 mL of + or – value of H_2_O_2_ according to Plackett-Burman design for 30 min. The cells and the supernatant were collected each as above. The cell pellets then washed by saline solution followed by centrifugation (as above). Finally, in the third step, the cell pellets were resuspended in 60% Ethanol and left at room temperature for 30 min with gentle vortex each five min for 30 sec. The cell pellets collection and washing were repeated as above. 

### 2.5. BGs Evaluation Using Light Microscope

 Bacterial smear for each treatment was prepared using standard criteria following by crystal violet staining. The cells from each experiment were examined by the aid of the light microscope. The quality of the cells has been determined based on the bacterial structure as either being correct or deformed and the overall BGQ given as %.

### 2.6. Determination of the DNA Concentration

 The concentration of the DNA was determined by measuring the absorption at 260 nm. Quartz cuvette was used. An extinction *E*_260_ = 1 corresponds to 50 *µ*g dsDNA mL^−1^ [16]. 

### 2.7. Determination of the Protein Concentration

 Protein analysis of each experiment (the different supernatants) was determined using the spectrophotometer at 280 nm. Quartz cuvette was used. The protein different concentration was derived from Bovine Serum Albumin standard curve. 

### 2.8. Sample Preparation for Electron Microscope Examination

For further study to the quality of BGs, electron microscope was used to scan the bacterial cells. Dry bacterial smear for each preparation was prepared and the smear surface then coated with approximately 15 nm gold (SPI-Module Sputter Coater).

### 2.9. Scanning of the BGs Surface

The golden coated sample then has been scanned by analytical scanning electron microscope (Jeal JSM-6360LA) with secondary element at 10 kv acceleration voltage at room temperature. The digital images then were adjusted and saved.

### 2.10. Liner Multiple Regression Analysis of Plackett-Burman Experiment

 The results obtained from Plackett-Burman design experiments were applied to linear multiple regression analysis using Microsoft Excel 2002 software. The linear multiple regression analysis was conducted for the BGQ as response. Variables with confidence levels bigger than, or equal to, 90% were considered significance. Variables whose confidence levels were less than 90% until 70% were considered as effective [12].

### 2.11. Generating 1st Order Model

The model created from the analysis of Plackett-Burman experimental design using linear multiple regression analysis is based on the 1st order model

(1) $$\begin{array}{c} Y=\beta_{o}+\Sigma \beta_{1}X_{i} \end{array}$$

(see [11]) where *Y* is the predicted response, *β*_*o*_ model constant, and *β*_1_ variable linear coefficient.

ANOVA test was generated for the response (BGQ) to determine the relationship among the variables at 90% or higher confidence level. 

### 2.12. Determination of the Variables Main Effect

 The main effect for each variable was determined from Table 1 using the following equation:

(2) $$\begin{array}{c} \text{main}\;\text{effect}=\sum \frac{\left(+1\right)}{n_{\left(+1\right)}}-\sum \frac{\left(-1\right)}{n_{\left(-1\right)}}. \end{array}$$

### 2.13. Determination of **E. coli ** Viability

 The various BG preparations were investigated for the possibility of the presence of any viable cells by subjecting them to grow in NA plats. Where 25 *µ*L from each sample was transferred to the surface of NA. The plats then were incubated at 37°C for five days. 

### 2.14. Lysis of the Viable Cells by Inducing the Lysis Gene on pLysS Plasmid

The lysis gene carried on the plasmid pLysS was induced by Chloramphenicol where the cells (which still being viable) were inoculated to NB was containing Chloramphenicol (50 *µ*g/mL) and incubated at 37°C and 100 rpm shaking rate for overnight. The NB then tested for the possibility of the existence of any viable cells after inducing the lysis gene by spreading 25 *µ*L over the surface of NA and incubating the plat(s) at 37°C for overnight as above. 

### 2.15. Agarose Gel Electrophoresis

 The existence of the genomic DNA or the plasmid was tested using agarose gel electrophoresis after lysis of the BGs using the slot lysis protocol. 1% agarose gel (Bioline, London, UK) containing 0.5 g/mL ethidium bromide was run in a horizontal gel electrophoresis unit (Mini-Sub DNA cell, BioRad). The running buffer was TAE (40 mM Tris, 20 mM acetic acid, 1 mM EDTA, pH 8.0). 1000 bp standard ladder was used (Promega, Madison, USA). Electrophoresis was carried out at 100 V for 1 h on an Amersham-Pharmacia Biotech (Uppsala, Sweden) power supplier unit ECPS3000/150. The stained bands were visualized with UV light (309 nm) using a transilluminator, and the gel was recorded as digital image using a gel documentation system (UVI-Tech). 

## 3. Results

### 3.1. Determination of MIC and MGC

The MIC of each of NaOH, SDS, and H_2_O_2_ was determined. In case of H_2_O_2_ the MIC was 40.8 *µ*L/mL from 30% H_2_O_2_. In case of SDS the MIC was 1.665 mg/mL. In case of NaOH the MIC was 0.0138 N. The determined MICs represent the + value in Plackett-Burman design. Another lower dosage has been determined for each of the above compounds to representing the – value in Plackett-Burman experiment and representing the first concentration, which allows the MGC, and they are 5.83 *µ*L/ml H_2_O_2_ from 30% H_2_O_2_, 0.00231 N NaOH, and 0.237 mg/mL of SDS. In case of CaCO_3 _the +1 was 1.05 *µ*g/mL while −1 was 0.35 *µ*g/mL.

### 3.2. Determination of **E. coli ** Viability

 Only experiment number 8 showed one single viable cell under the experimental condition as described in the Material and Methods section.

### 3.3. Plackett-Burman Experiments

 The data in Table 1 show that Plackett-Burman experimental design succeeded to randomize the variables to get the proper results. The BGQ was used as a response. The results were determined as percentage. Experiments number one and eleven show 100% BGQ. In Table 1 the amounts of the released DNA and protein were located in the basic experiment (first step), while minor release was shown in both of H_2_O_2_ and Ethanol treatment steps (second and third steps). 

### 3.4. Linear Multiple Regression Analysis of Plackett-Burman Experiments

 The results obtained from the Plackett-Burman twelve experiments (Table 1) were analyzed using linear multiple regression analysis statistical method. From the regression analysis, the best-fitted model was generated. ANOVA test was conducted to determine the level of significance. The estimate, standard error, *T* statistic, *P* value, and Confidence level percentage for each of the variables (CaCO_3_, H_2_O_2_, NaOH, SDS, and shaking rate/temperature) were calculated and represented in Table 3.

The NaOH has negative effect with confidence level equal to 99.64%. NaOH has the highest negative effect on the BGQ. H_2_O_2_ has confidence level equal to 69.76%, which could be considered as effective positively in the BGQ. Each of CaCO_3_, SDS, and shaking rate/temperature has a confidence level equal to 58.59%, 9.56%, and 44.6%, respectively, which considered to be noneffective on the BGQ. 

The fitting multiple linear regression model describes the relationship between BGs quality (BGQ) and five independent variables. The equation of the fitted model is

(3) BGQ=55.8333−5.83333∗CaCO3+7.5∗H2O2−30.8333∗NaOH−0.833333∗SDS−4.16667∗(Shaking  rate  and  Temperature).

Since the *P* value in the ANOVA Table 4 is less than 0.05, there is a statistically significant relationship among the variables at the 95.0% confidence level. The *R*-squared statistic shows that the model as fitted explains 79.9685% of the variability in BGQ. The adjusted *R*-squared statistic is 63.2757%. The standard error of the estimate shows the standard deviation of the residuals to be 23.0338. The mean absolute error (MAE) of 13.8889 is the average value of the residuals. The Durbin-Watson (DW) statistic tests the residuals to determine if there is any significant correlation based on the order in which they occur in the data file. Since the *P* value is greater than 0.05, there is no indication of serial autocorrelation in the residuals at the 95.0% confidence level. 

**Figure 1:**
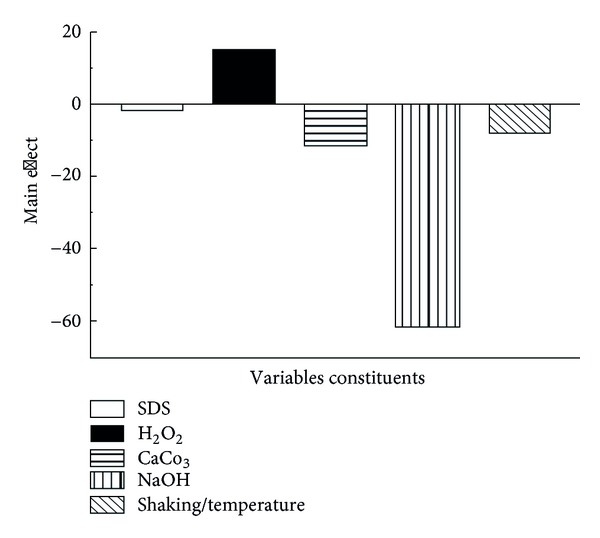
Main effect of the five used variables.

In both of the experiments number one and eleven NaOH was in its −1 value, that is, agrees with our conclusion from the main effect in Figure 1. NaOH is negatively affecting the cell quality if used in its +1 value.

The main effect as in Table 1 and Figure 1 shows that all variables except H_2_O_2_ have negative effect on the cells quality. This is logic where SDS, CaCO_3_, and NaOH are well known for their ability to impair the cell wall. However, only NaOH shows a significant negative effect. Shacking rate and temperature also have minor effect on the cell quality. H_2_O_2_ according to the main effect analysis as in Table 2 has a positive effect on the cells quality. However, it might be an existence of unknown side effect on the macromolecule, which should be investigated in future studies. 

## 4. Discussion

The applications of BGs was recevied recently an increasing interest especially those concerning the medical and pharmaceutical applications [3–7]. In this study, a protocol other than *E* lysis gene for BGs preparation was introduced. The protocol depends on growing the bacterial cells in condition allowing a correct cell wall formation then subjecting the cells to chemicals that could affect the cell wall in a ways enables forming BGs. By controlling the concentration of those chemical compounds, one can introduce the best effect, which matches our target. For more precise determination for such concentration, the MIC and the MGC of each of NaOH, H_2_O_2_, and SDS was determined. MIC will give us the minimum quantity of the used chemicals responsible for the bacterial killing. MGC will give the minimum concentration, which allows the bacterial cells to survive. While vegetative cells are more sensitive to those used chemical compounds, we have used a fully grown and a three-day age cells which are expected to have more resistant cell wall which means that the quantity used will have less effects on the cell wall quality. Moreover, we use the MGC, which already allow those cells to escape and live in the existence of those chemical compounds while it must still have an effect on the cell wall. While we use more than one variable to affect the cell wall of the bacteria, the multiple variables randomization and optimization will be the proper choice to optimize the process of BGs preparation. Using Plackett-Burman design to randomize those variables allows us to map the best conditions where the cells can be evacuated from their DNA and protein content [11, 12]. Such release of the protein and DNA can be monitored simply by the aid of the spectrophotometer. Also, it could be an indicator of the efficacy of each experiment. Spectrophotometer was used at 260 and 280 nm to determine the DNA and the protein amounts, respectively, which released during each step of preparation. High release of each of the DNA and the protein will indicate that the cells lose their content of them both and turned to BGs. However, complete cells lysis should be avoided. The cells were harvested and washed by saline (0.5%) to remove any debris, fatty materials, DNA/protein, and the residue of the used chemicals. A sample was taken from each experiment and cultivated on NA plat for long time (five days) at 37°C to investigate the existence of any viable cells.

**Figure 2:**
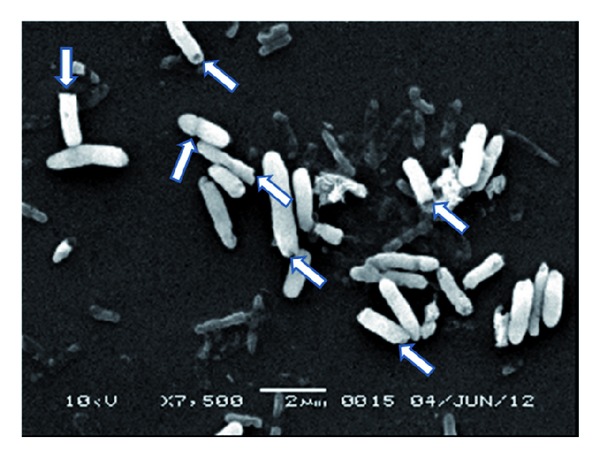
Scanning electron microscope for *E. coli* BG cells. Arrows show the pores in some of the *E. coli* BG cells.

**Figure 3:**
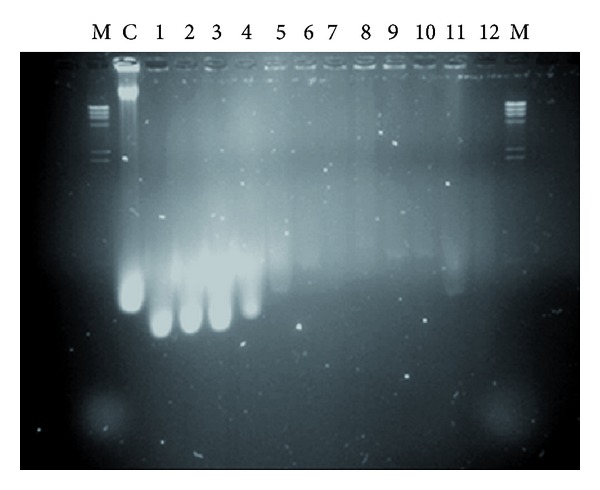
AGE for the marker (M), control (C), and twelve experiments (1–12).

Each experiment started with incubating the cells with SDS, NaOH, and CaCO_3_ for 1 hr; then the cell washed by 0.5% saline and then incubated with H_2_O_2_ followed by cell washing with 0.5 saline. The amount of the DNA and protein, which released from the cells after treatment with NaOH, SDS, and CaCO_3_ was determined (first step). In addition, the released amount of DNA and protein was determined after treatment with H_2_O_2_ (second step). The cell quality for each experiment was determined by using light microscope as percentage. The quality of the final prepared BGs was determined using scanning electron microscope (Figure 2). Figure 2 shows that the *E. coli* BGs have each a tiny small pore which is responsible for the evacuation of the cells from their contents of the macromolecules. The cells were in good quality and maintain their 3D structure. BGQ was used as a main response. The main effect of each variable from the twelve experiments in Table 1 was determined. It is important to highlight that the main effect analysis is an important tool for monitoring the effect of each variable regarding its used concentrations (+1 and −1) as shown in Figure 1 and Table 2. The main effect can give a fast signal about what is going on. In our case, the concentrations, which we used, are homogenous except NaOH, which significantly has a negative effect on the BGQ as proved by the main effect analysis (Figure 1). However, this effect is mainly because of the +1 concentration, while in experiments number one and eleven, which give an excellent BGQ, the −1 concentration was used. The logical analysis of the data should be passing side by side with the statistical analysis. Moreover, the final content of the bacterial DNA for each experiment was evaluated using AGE (Figure 3). The AGE as in Figure 3 shows perfectly that the DNA was mostly degraded. In Figure 3 we can observe that the genomic and the plasmid DNA have been completely degraded comparing with the control. Even so, some lines show nearly no DNA. 

Plackett-Burman design succeeded to map the points where the cells could be perfectly evacuated from their protein and DNA. The SEM proves that the cells are in good conditions and still have the correct 3D shape. This protocol also included the use of the cells lysis lysozyme gene, which degrades any viable cells existed. This might find some interest in applications such as the preparation of fragmented BGs. However, only one experiment out of 12 experiments shows the existence of viable cells. The AGE shows that all the genetic materials were degraded. In fact, this protocol is a collection of the experiences gained from many other protocols where CaCO_3_ was selected from the competent cells preparation protocol, which allows plasmid transformation without impairing the cell viability. SDS is able to degrade the cell wall; however, if used in appropriate amount it could be used for our aim. H_2_O_2_ has oxidizing activity and able to degrade the genetic material instead of using nucleases [2]. NaOH is also known for their effect in the cell wall.

## 5. Conclusion

This protocol is designed to use cheap and safe chemical compounds for BGs preparation. Each of NaOH, SDS, H_2_O_2_, and CaCO_3_ was used. The MIC and MGC for each was determined and used as +1 or −1 in twelve experiments represent Plackett-Burman design. This method was used to map the proper conditions for BGs preparation. The BGQ was determined by the aid of each of the light and electronic microscope. The amounts of the DNA and protein released during each step were determined. The protocol concerns not only BGs preparation but also detecting any still viable cells and their lysis. This protocol, the used chemical compounds, could be used as a general protocol for BGs preparation from other microbial strains.

## Figures and Tables

**Table 1:** Plackett-Burman experimental design.

Exp. no.	Variables					Basic Experiment	Basic Experiment	H_2_O_2_	H_2_O_2_	Ethanol	Ethanol	Response
	SDS	H_2_O_2_	CaCO_3_	NaOH	Shaking rate/Temperature	DNA *µ*g/mL	Protein *µ*g/mL	DNA *µ*g/mL	Protein *µ*g/mL	DNA *µ*g/mL	Protein *µ*g/mL	BGQ%
1	1	1	1	−1	1	164.5	3425.445	20.2	353.547	8.1	45.477	100
2	−1	−1	−1	−1	−1	136.2	2400.012	16.45	432.765	11.5	500.247	90
3	1	−1	1	1	−1	178.6	3137.913	0.6	0	16.55	391.689	10
4	−1	−1	−1	1	1	74.2	2589.255	4.4	151.101	5.65	123.228	30
5	−1	1	1	1	−1	121.15	2078.739	19.25	337.41	9.7	38.142	50
6	1	1	−1	1	−1	99.85	3082.167	16.1	222.984	5.4	233.253	60
7	−1	1	−1	−1	−1	106.1	1880.694	33	651.348	4.75	126.162	90
8	−1	−1	1	1	1	146.9	2400.012	33	284.598	7.7	29.34	0
9	1	1	−1	1	1	0	0	5.15	968.22	123.6	4.401	0
10	−1	1	1	−1	1	90.15	1805.877	13.1	1043.037	6.8	36.675	80
11	1	−1	−1	−1	1	122.2	2100.744	12.05	355.014	5.05	36.675	100
12	1	−1	1	−1	−1	59.4	2565.783	8.15	234.72	6.75	365.283	60

**Table 2:** Main effect of each variable.

Variable	Values		Main effect
	(∑+1)/*n*_(+1)_	(∑−1)/*n*_(−1)_	∑(+1)/*n*_(+1)_ − ∑(−1)/*n*_(−1)_
SDS	55	56.66667	−1.66667
H_2_O_2_	63.33333	48.33333	15
CaCO3	50	61.66667	−11.6667
NaOH	25	86.66667	−61.6667
Shaking rate/temperature	51.66667	59.83333	−8.16667

**Table 3:** Linear multiple regression analysis of Plackett-Burman.

Parameter	Estimate	Standard error	*T* statistic	*P* value	Confidence level %
CONSTANT	55.8333	6.64928	8.3969	0.0002	99.98
CaCO_3_	−5.83333	6.64928	−0.877288	0.4141	58.59
H_2_O_2_	7.5	6.64928	1.12794	0.3024	69.76
NaOH	−30.8333	6.64928	−4.63709	0.0036	99.64
SDS	−0.833333	6.64928	−0.125327	0.9044	9.56
Shacking rate/temperature	−4.16667	6.64928	−0.626634	0.5540	44.6

**Table 4:** ANOVA test.

Source	Sum of squares	Df	Mean square	*F*-ratio	*P*-value
Model	12708.3	5	2541.67	4.79	0.0414
Residual	3183.33	6	530.556		

Total (Corr.)	15891.7	11			

*R*-squared = 79.9685 percent; *R*-squared (adjusted for d.f.) = 63.2757 percent; standard error of Est. = 23.0338; mean absolute error = 13.8889; Durbin-Watson statistic = 1.6178 (*P* = 0.3319); lag 1 residual autocorrelation = 0.103839.
